# Spatial and temporal coevolution of N2 neuraminidase and H1 and H3 hemagglutinin genes of influenza A virus in US swine

**DOI:** 10.1093/ve/veab090

**Published:** 2021-10-08

**Authors:** Michael A Zeller, Jennifer Chang, Amy L Vincent, Phillip C Gauger, Tavis K Anderson

**Affiliations:** Department of Veterinary Diagnostic and Production Animal Medicine, College of Veterinary Medicine, Iowa State University, 1800 Christensen Drive, Ames, IA 50011, USA; Bioinformatics and Computational Biology Program, Iowa State University, 2014 Molecular Biology Building, Ames, IA 50011, USA; Virus and Prion Research Unit, National Animal Disease Center, USDA-ARS, 1920 Dayton Avenue, Ames, IA 50010, USA; Virus and Prion Research Unit, National Animal Disease Center, USDA-ARS, 1920 Dayton Avenue, Ames, IA 50010, USA; Department of Veterinary Diagnostic and Production Animal Medicine, College of Veterinary Medicine, Iowa State University, 1800 Christensen Drive, Ames, IA 50011, USA; Virus and Prion Research Unit, National Animal Disease Center, USDA-ARS, 1920 Dayton Avenue, Ames, IA 50010, USA

**Keywords:** neuraminidase, genomic epidemiology, influenza A virus, swine, vaccine

## Abstract

The neuraminidase (NA) and hemagglutinin (HA) are essential surface glycoproteins of influenza A virus (IAV). In this study, the evolution of subtype N2 NA paired with H1 and H3 subtype HA in swine was evaluated to understand if the genetic diversity of HA and NA were linked. Using time-scaled Bayesian phylodynamic analyses, the relationships of paired swine N2 with H1 or H3 from 2009 to 2018 were evaluated. These data demonstrated increased relative genetic diversity within the major N2 clades circulating in swine in the USA (N2.1998 between 2014 and 2017 and N2.2002 between 2010 and 2016). Preferential pairing was observed among specific NA and HA genetic clades. Gene reassortment between cocirculating influenza A strains resulted in novel pairings that persisted. The changes in genetic diversity in the NA gene were quantified using Bayesian phylodynamic analyses, and increases in diversity were observed subsequent to novel NA–HA reassortment events. The rate of evolution among NA–N2 clades and HA–H1 and HA–H3 clades were similar. Bayesian phylodynamic analyses demonstrated strong spatial patterns in N2 genetic diversity, but frequent interstate movement of rare N2 clades provided opportunity for reassortment and emergence of new N2–HA pairings. The frequent regional movement of pigs and their influenza viruses is an explanation for the documented patterns of reassortment and subsequent changes in gene diversity. The reassortment and evolution of NA and linked HA evolution may result in antigenic drift of both major surface glycoproteins, reducing vaccine efficacy, with subsequent impact on animal health.

## 1. Introduction

Influenza A virus (IAV) is an important respiratory pathogen with high economic consequences in commercial swine production systems with a risk for zoonotic transmission (Vincent et al. 2008b; Dykhuis-Haden et al. 2012). Clinical signs of IAV in swine include cough, fever, lethargy, and a reduction of appetite that may lead to weight loss. IAV-induced lung pathology often predisposes swine to secondary bacterial infections, resulting in further production losses and increased risk for mortality (Gramer 2006; Wang et al. 2013). Due to the high morbidity associated with clinical disease, prevention and control of IAV are necessary to minimize animal suffering, mitigate production loss, and protect public health.

Vaccines have historically been formulated based on the hemagglutinin (HA) protein, which is the primary target of protective immune responses (Sandbulte et al. 2015; Vincent et al. 2017). Vaccines that contain HA antigens that are well-matched to the genetic diversity of circulating IAV in swine have demonstrated efficacy, but this is greatly reduced when applied to control antigenically drifted IAV (Vincent et al. 2008a). Further, the H1 and H3 HA subtypes of IAV that are detected in US swine contain at least 14 genetic clades with distinct antigenic properties (Anderson et al. 2013; Zeller et al. 2018a). Consequently, a potential strategy to increase the breadth of protection offered by IAV vaccines is the inclusion of additional proteins. Research has suggested that the neuraminidase (NA) gene may also contribute to vaccine efficacy through NA inhibiting antibodies (Monto and Kendal 1973; Sandbulte et al. 2016).

N1 and N2 subtype NA genes circulate in the USA, with the N2 subtype being detected in approximately two-thirds of swine IAV reported in surveillance programs (Anderson et al. 2013; Zeller et al. 2018a). Within these NA subtypes, there are two N1 genetic clades, including the classical swine lineage (N1.Classical), which emerged coincident with the 1918 H1N1 introduction into swine (Koen 1919), and a Eurasian swine lineage N1 gene (N1.Pandemic) that emerged in the USA associated with the 2009 H1N1 human pandemic (Dawood 2009; Smith et al. 2009). Contemporary N2 genetic clades circulating in swine are derived from human-to-swine transmission episodes: the first associated with human seasonal IAV in the late 1990s (N2.1998) and the second in the early 2000s (N2.2002). The N2.1998 lineage was introduced into swine with the triple reassortant IAV that evolved into the H3.Cluster IV HA (Zhou et al. 1999). The N2.2002 lineage was introduced during a human to swine spillover coincident with the H1.Delta clades (Vincent et al. 2009). Although veterinary diagnostic labs continue to occasionally detect sporadic human seasonal NA in swine (Zeller et al. 2018b), these have yet to become widely established.

The quantification of genetic and antigenic diversity described in IAV in swine has largely focused on the HA gene. However, the NA and HA function in a coordinated effort to replicate within and transmit between hosts. Functionally, a balance in NA and HA cellular interactions is necessary for IAV infection to result in successful host-to-host transmission (Mitnaul et al. 2000; Das et al. 2011). Links between these genes have been documented in phylogenetic studies on human seasonal H3N2, which have suggested that substitutions in one gene affect substitutions in the other (Neverov et al. 2014). In swine, a similar type of evolutionary dynamic is plausible given the 14 different HA genetic clades and 4 NA lineages with considerable genetic diversity within each, but the degree to which this affects observed diversity is unknown.

The objective of this study was to assess the NA genetic diversity of IAV circulating in US swine and evaluate how evolution and reassortment pairing new HA subtypes or clades to these N2 genes may affect genetic diversity and evolutionary trajectories. This research demonstrates that the genetic diversity of N2 circulating in swine within the USA has increased. Analysis of pairing between HA and NA clades strongly suggests that this pairing is not random, and that underlying factors may limit viable NA–HA pairs. Subsequent to reassortment, increases or decreases in the genetic diversity of HA-NA pairs are correlated, although further work is needed to discern the underlying mechanism. Understanding how HA pairing and reassortment impacts the evolutionary dynamics and extent of genetic diversity of the NA in swine IAV is critical to understanding how genomic context affects the emergence and transmission of IAV in swine and will facilitate control efforts that include NA in vaccine strain selection.

## 2. Materials and methods

### 2.1 Data acquisition and phylogenetic clade assignment

Approximately 4,000 paired nucleotide N2 NA and HA sequences detected in swine with accompanying metadata were downloaded from the Influenza Research Database (IRD) on 18 October 2018 (Squires et al. 2012; Yun Zhang et al. 2017). Data were limited to IAV swine sequences from the USA from 9 August 2009 to 20 September 2018. All NA sequences less than 1,200 nt were removed (representing less that 85 per cent of the coding region). Sequences associated with agricultural fair events and pigs were removed as they do not represent IAV from the general commercial swine population (i.e. sequences annotated with ‘OSU’) (Bowman et al. 2017).

Phylogenetic clade classifications for H1 viruses were designated using the Swine H1 Clade Classification Tool provided on the IRD (Anderson et al. 2016). H3 and NA clades were determined through maximum-likelihood phylogenetic analysis using reference sequences (Chang et al. 2019). Reference nucleotide sequences and downloaded IAV from swine were aligned using MAFFT v7.27 (Katoh and Standley 2013). Maximum-likelihood trees were inferred using FastTree2 v2.1.9 (Price, Dehal, and Arkin 2010) for each gene alignment using a general time reversible (GTR) model of nucleotide substitution with each IAV gene classified to clade based upon the nearest neighbor in the reference gene dataset. Within and between-clade distances were calculated using MEGA X v10.1 (Kumar et al. 2018).

### 2.2 Quantifying relative genetic diversity in swine IAV N2 NA genes

Estimates of relative diversity for the N2.2002 and N2.1998 genetic lineages were determined through time-scaled Bayesian phylodynamic analyses and the inference of effective population size (EPS). Based upon computational limitations, we randomly sampled 620 genes from the N2.2002 lineage (from *n* = 3,650 total genes). These genes were aligned using MAFFT v7.27 (Katoh and Standley 2013) and a maximum-likelihood tree was inferred using FastTree2 v2.1.9 (Price, Dehal, and Arkin 2010). Root-to-tip divergence was analyzed using TempEST v1.5.1 (Rambaut et al. 2016) and genes with incongruent divergence and sampling date were removed (data available at https://github.com/flu-crew/n2-diversity/tree/master/N2.2002_eps). The resulting final dataset consisted of 600 nonidentical N2.2002 genes. All of the available N2.1998 genes (*n* = 596 taxa) were aligned, those that were not at least 50 per cent of the gene were removed, and a maximum-likelihood tree was inferred with root-to-tip divergence analyzed in TempEST v1.5.1 with two taxa removed as outliers, resulting in 583 taxa for analysis (data available at https://github.com/flu-crew/n2-diversity/tree/master/N2.1998_eps).

These datasets were analyzed using Bayesian phylogenetic methods in BEAST v1.8.4 (Drummond et al. 2012) with the BEAGLE library v3.1.2 (Ayres et al. 2011), implementing a generalized time reversible (GTR) nucleotide substitution model (Tavaré 1986) with gamma-distributed site heterogeneity (Yang 1994). We employed an uncorrelated relaxed clock with lognormal distribution (Drummond et al. 2006), and a Gaussian Markov random field (GMRF) Bayesian skyride with time aware-smoothing as the coalescent model (Minin, Bloomquist, and Suchard 2008). The Markov chain Monte Carlo (MCMC) chain length was set to 100 million iterations with sampling every 10,000 iterations. The results were analyzed using the GMRF skyride reconstruction in Tracer v1.6 (Rambaut and Drummond 2007). Additionally, time-scaled maximum clade credibility (MCC) trees were generated using TreeAnnotator v1.8.4 using median node heights and 10 per cent burn-in (Drummond et al. 2012). Dates of time to the most recent common ancestor (TMRCA) were inferred from the nodes between clades, using the 95 per cent higher posterior density (HPD) as the range of uncertainty.

### 2.3 Measuring concurrent changes in the diversity of conserved NA and HA gene pairings

The frequency of particular NA and HA pairings was measured by splitting the data into the N2.2002 and N2.1998 monophyletic clades. NA–HA pairings that did not occur more than 10 times were removed from the analysis, and the significance of gene pairings was measured with a post hoc analysis following Pearson’s chi square test for independence using the standard residuals in R v3.3.3 (R Core Team 2015). A confirmatory Fisher’s exact test was performed due to sparsity of some NA–HA pairings.

Of these documented NA and HA pairings, N2.1998B was almost exclusively paired with H1.Delta2 HA genes (449 of 462 genes). Consequently, we analyzed the evolutionary dynamics of 462 taxa of N2.1998B clade NA genes and 522 taxa of H1.Delta2 HA genes separately. These genes were aligned, poor quality data were removed, and root-to-tip divergence was analyzed, with divergent sequences removed. This process resulted in 458 N2.1998B genes and 517 H1.Delta2 genes (data available at https://github.com/flu-crew/n2-diversity/tree/master/H1.delta2_eps). These data were analyzed using BEAST v1.8.4 implementing a GTR substitution model with gamma-distributed rate variations, an uncorrelated relaxed clock with lognormal distribution, and the GMRF Bayesian skyride coalescent. The MCMC chain was run for 100 million iterations with sampling every 10,000 iterations. The relative genetic diversity for both clades N2.1998B and H1.Delta2 was plotted to examine changes in the EPS. A similar analysis with the same analytical settings was conducted on the N2.2002A genes that were paired with H1.Delta1B HA (following screening, *n* = 842 N2.2002A and H1.Delta1B taxa for analysis with data available at https://github.com/flu-crew/n2-diversity/tree/master/H1.delta1b_eps).

### 2.4 Estimating the evolutionary rate of swine IAV N2 NA and HA genes

To estimate the evolutionary rate of N2 NA, we created alignments of each of the major of genetic clades: N2.1998A, N2.1998B, N2.2002A, and N2.2002B (*n* = 115, 458, 262, 310, respectively). To estimate the evolutionary rate of H1 and H3 HA, we generated alignments of H1.Gamma, H1.Delta2, H3.2010.1, and H3.ClusterIVA (*n* = 300, 300, 299, 298). Using BEAST 2 v2.5.1 (Bouckaert et al. 2014), we implemented an unlinked codon substitution model split into independent partitions (1 + 2 + 3) and unlinked clock models for each codon position. The analysis used a GTR nucleotide substitution model with a gamma site heterogeneity, an uncorrelated relaxed clock with lognormal distribution, and an exponential population growth coalescent tree prior. The MCMC chain length was set to 100 million iterations with sampling every 10,000 iterations. The mean rate of substitution was calculated by averaging the values for the three codon position’s mean rate of substitution in terms of sites/substitution/year with distribution for each and visualized in R v3.3.3 using the ggplot2 package (Wickham 2009).

### 2.5 Detecting diversification following reassortment of N2 NA in swine IAV

Reassortment in the N2 NA gene was identified using phylogenetic incongruence in gene trees (Boni et al. 2010). Maximum-likelihood trees were inferred for N2.1998 and N2.2002 using RAxML v8.2.11 (Stamatakis 2014) with a GTR model with a gamma-distributed rate variation and statistical support values determined using rapid bootstrapping with the autoMRE criterion. Trees were visualized in FigTree v1.4.3, and we defined sustained transmission of a reassorted pairing as one where a monophyletic clade of NA genes acquired a novel HA genetic clade, and the NA–HA combination was maintained within the monophyletic clade for more than 10 subsequent branches. To facilitate visualization, a randomly sampled set of 300 HA and NA genes were sorted and transformed into proportional branch length cladograms and built into a tanglegram in R v3.3.3 (R Core Team 2015) using the dendextend v1.9.0 package (Galili 2015). Branches on these constituent maximum-likelihood trees were colored by phylogenetic clades with lines connecting trees colored based on HA clade to emphasize reassortment events.

The mean rate of substitution of NA was compared to assess whether reassortment affected the substitution rate. To achieve this, we identified and extracted monophyletic clades that contained a reassortment event. Extracted sequence data for each identified clade were realigned using MAFFT v7.27 (Katoh and Standley 2013), and an MCC tree for each alignment was inferred using BEAST 1.8.4 (Drummond et al. 2012). Data were partitioned based on whether the NA was paired with the donor HA, or the recipient HA of the reassortment event with unlinked clock models and substitution models and a GTR nucleotide substitution model (Tavaré 1986) with gamma-distributed site heterogeneity (Yang 1994). We employed an uncorrelated relaxed clock with lognormal distribution (Drummond et al. 2006) and an exponential growth coalescent model (Drummond et al. 2002). The MCMC chain length was set to 100 million iterations with sampling every 10,000 iterations. The mean substitution rate distribution was extracted for each partition with the initial 10 per cent discarded as burn-in. Some prior and posterior parameters never fully converged and had relatively low estimated sampling sizes due to the small alignments analyzed; however, the likelihood and meanRate parameters that were important in the analysis in all cases converged with ESS > 200. The credible intervals of the distribution of the partitioned meanRate parameters were compared using Bayesian estimation methods developed to assess group means and their differences (Kruschke 2013). This approach assessed the posterior distributions of pre- and post-reassortment substitution rates: the two distributions were subtracted from each other, and the 95 per cent HPD (credible interval) was recalculated using the HPD function from the BayesTwin package in R (Schwabe 2017). If zero was included in the credible interval, the two substitution rates were deemed to be equivalent. If zero was outside the credible interval, then the distributions of mean substitution rates had nonoverlapping credible intervals and were deemed nonequivalent. A time-scaled MCC trees were generated using TreeAnnotator v1.8.4 using median node heights and 10 per cent burn-in (Drummond et al. 2012). The time of reassortment was estimated by identifying the node of the most recent common ancestor for the clade with the new NA–HA gene pairing.

Within the NA genes, we used mixed-effects model of evolution (MEME) (Murrell et al. 2012), part of the HyPhy package v2.3.14, to test for instances of episodic and pervasive selection by using an MEME. The results from MEME were confirmed by conducting concurrent analyses with the Fixed Effects Likelihood (FEL) and Single-Likelihood Ancestor Counting (SLAC) methods (Kosakovsky Pond and Frost 2005) and reviewed via HyPhy vision v2.4.3. Selective sweeps were tested and visualized using Sweep Dynamics plots (Klingen et al. 2018). To facilitate interpretation, 42 ‘N’ nucleotide ambiguities were inserted at the 5ʹ-end of each NA sequence and the length of the signal peptide was set to 14 when running the Sweep Dynamics plots docker instance to allow the start methionine to be labeled as position 1 in the output. Sweeps that exceeded the detection of 50 per cent within any yearly influenza season were logged and presented. Each N2 genetic clade was visualized using a local Nextstrain (Hadfield et al. 2018) implementation, with mutations annotated across the phylogenetic backbone alongside mutations identified through the Sweep Dynamics plots.

### 2.6 Inferring permissiveness of N2 to HA gene pairing in swine IAV

To quantitatively characterize the exchange of NA and HA genes, we adapted a phylogeographic approach, using NA genetic clade information as our ‘location state’. The same data subset and setup were used as in our analyses on quantifying changes in relative genetic diversity. Each NA gene had an HA genetic clade designated as a trait, and transitions from one category to another (e.g. from N2.1998B/H1.Gamma to N2.1998B/H3 2010.1) were inferred along the internal branches representing the evolutionary history of the virus. These transitions are termed Markov jumps and state change counts for the HA clade trait were reconstructed in BEAST v1.8.4. The sites for the trait were calculated using an asymmetric substitution model, a Bayesian stochastic search variable selection (Edwards et al.), and an uncorrelated relaxed clock model with lognormal distribution. Analysis output was assessed in Tracer v1.6.0 to confirm convergence, and SPREAD3 v0.9.6 (Bielejec et al. 2016) was used to compute the Bayes factors with 10 per cent burn-in. Bayes factors were plotted using the inferno color scheme from the R viridis package v0.5.1 using the ggplot2 v3.1.0 and cowplot v0.9.4.

### 2.7 Assessing the spatial distribution of N2 NA genes in swine IAV

Swine IAV N2 data were reduced to subsets that included only sequences that included a US state. The number of detections of each N2 genetic clade was rendered on a US map using the R maps module v3.3.0, colored to emphasize differences using the viridis color scheme and rendered by the ggplot2 v3.1.0 package. Statistical association between the N2 genetic clade and state of collection was assessed with Pearson’s chi square test for independence, and then a post hoc analysis of the chi square test using the standard residuals in R v3.3.3 (R Core Team 2015). A Fisher’s exact test was performed to confirm the results of the initial chi square test. To quantify the movement of the NA genes across the US, we used a phylogeographic approach with the US state as our ‘location state’. Each NA gene had a US state designated as a trait, and transitions from one category to another (e.g. from North Carolina to Iowa) were inferred along the internal branches representing the evolutionary history of the virus. These number of state change counts for the US state trait were reconstructed in BEAST v1.8.4 following the same methods described above.

## 3. Results

### 3.1 Increased N2 relative genetic diversity

**Figure 1.**
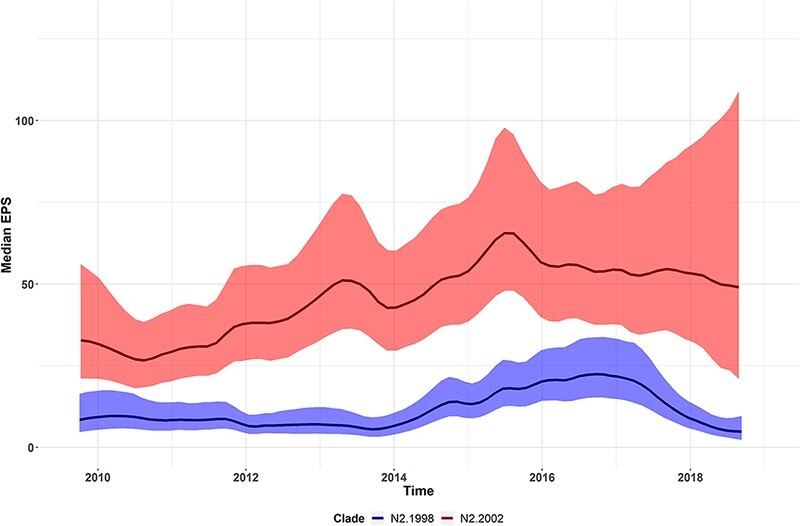
Relative genetic diversity, measured as EPS, of the predominant N2 NA clades in IAVs detected from 2009 to 2018 in swine in the USA (N2.1998 in blue and N2.2002 in red). Median EPS denoted by solid colored lines with the 95 per cent HPD shaded in the same color. Relative genetic diversity increased linearly in the N2.1998 between 2014 and 2017 and between 2010 and 2016 in the N2.2002.

Four statistically supported monophyletic clades were named within the broader swine N2 lineages (Fig. 3). These clades were named due to detection frequency (*n* > 10 genes collected from multiple locations across multiple years), statistical support (posterior probability of 1), and average within-clade and between-clade diversity of <5 per cent and >5 per cent, respectively. The TMRCA for the N2.1998A and N2.1998B clades was 2007.6 (2006.5–2008.8 95 per cent HPD), and the TMRCA for the N2.2002A and N2.2002B clades was 2006.0 (2004.9–2007.1, 95 per cent HPD). For each of the N2.1998 and N2.2002 monophyletic clades, the average within-clade distance was 3.1 per cent and the between-clade distance was greater than 6 per cent (Table 1). There was clade-specific variation in relative genetic diversity (Fig. S1). Changes in relative genetic diversity in swine IAV N2 lineages and clades were quantified by the EPS. The N2.1998 demonstrated an increase in diversity between 2014 and 2017, whereas the N2.2002 lineage demonstrated an increase in diversity between 2010 and 2016 (Fig. 1). The N2.1998A had a constant EPS, compared to the increasing EPS observed in the N2.1998B clade. The N2.2002A demonstrated an initial increase that peaked in mid-2012, before being gradually superseded by the N2.2002B by mid-2014 and peaking in 2015. These data suggest a general trend of increasing relative genetic diversity observed in both N2 lineages. The N2.1998B clade appears to be the major driver of the increased EPS of the N2.1998 lineage, whereas the N2.2002A and N2.2002B demonstrated different temporal trends that both contributed to the overall increase in EPS across the study period.

**Table 1.** Average percentage pairwise nucleotide distances within and between the statistically supported monophyletic NA N2 genetic clades. The average within-clade pairwise nucleotide distance was 3.1 per cent, and the average between-clade nucleotide distance was 9.5 per cent.

	N2.1998	N2.1998A	N2.1998B	N2.2002	N2.2002A	N2.2002B
N2.1998	2.8%					
N2.1998A	6.9%	2.5%				
N2.1998B	7.9%	11.4%	3.5%			
N2.2002	7.2%	9.8%	11.3%	2.6%		
N2.2002A	8.8%	11.8%	12.2%	6.1%	3.7%	
N2.2002B	9.3%	11.8%	13.6%	6.5%	8.0%	3.7%

**Figure 2.**
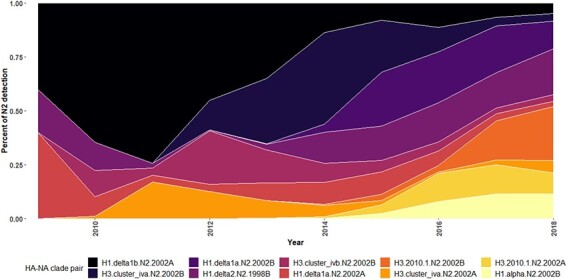
Temporal patterns of the 10 major HA and NA pairings detected in US swine from August 2009 to July 2018.

**Figure 3.**
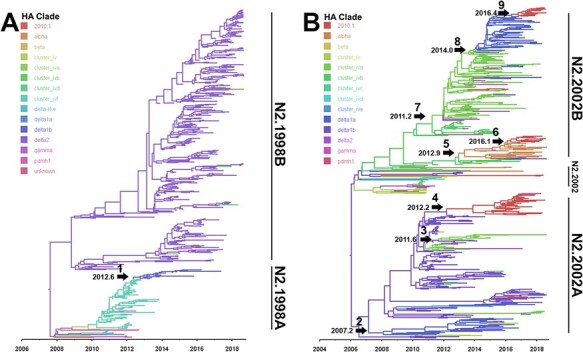
Phylogenetic relationships of the (A) N2.1998 and (B) N2.2002 lineage NA genes with N2 sequences detected in US swine from August 2009 to July 2018. Presented are time-scaled Bayesian MCC trees for each major N2 lineage and genetic clade with tree branches colored by HA genetic clade. A numbered arrow indicates a reassortment event and inferred date of the event where an N2 gene was paired with a new HA genetic clade with subsequent sustained detection in the swine population (>10 detections). The nine numbered arrows represent HA gene exchanges: (1) H3 C-IVF to H1 Delta1B; (2) H1 Delta1B to H1 Delta1A; (3) H1 Delta1B to H3 C-IVA; (4) H1 Delta1B to H3 2010.1; (5) H3 C-IVB to H1 Alpha; (6) H1 Alpha to H3 2010.1; (7) H3 C-IVB to H3 C-IVA; (8) H3 C-IVA to H1 Delta1A; and (9) H1 Delta1A to H3 2010.1. Each sustained reassortment event demonstrated increased genetic divergence, visualized as longer branch lengths in the phylogeny. The phylogenies with tip labels and posterior probabilities included are available at https://github.com/flu-crew/n2-diversity.

### 3.2 N2 and HA reassortment and linkage disequilibrium

NA–HA pairing was inferred from the N2 trees annotated by the HA clade. The 10 most frequently observed NA–HA pairs were identified, and their detections over time are shown in Fig. 2: these data demonstrate concurrent circulation and temporal dominance of NA–HA pairings that change yearly. Temporal dominance of different NA–HA pairings also varied across geographic regions (Fig. S2), with some regions maintaining no more than three major pairings (North Carolina, Illinois, and Indiana), whereas others maintained 7–10 pairings in almost equal numbers (Iowa, Minnesota, and Nebraska). Distinct clustering of HA clade groups was evident, along with shifts in HA clade annotation on the NA genes as a likely result of reassortment (Fig. 3). We defined establishment of the IAV HA and N2 clade pairing within the swine population when they were observed with greater than 10 detections across multiple years. Nine such reassortment events were found and annotated (Fig. 3). A Bayesian analysis was used to statistically validate if there was a change in the rate of substitution of the N2 NA before and after HA and NA reassortment (Fig. S3). Of the nine reassortment events observed, four had evolutionary rates pre- and post-reassortment that were significantly different (i.e. the mean substitution rates were different). The reassortment event labeled as ‘1’ in Fig. 3A, a transition in N2.1998A from H3.ClusterIVF to H1.Delta1B, showed that the rate of substitution slowed following reassortment, from 0.57 to 0.0036 substitutions/site/year (Fig. S3A). However, this result may be driven by sampling artifacts as the substitution rates of the NA genes paired with H3.ClusterIVF were unusually high and outside the bounds we document for analyses across entire genetic clades (Fig. S4). The reassortment event labeled as ‘7’ in Fig. 3B described an N2.2002B paired with H3.ClusterIVB reassorting to Cluster IVA: in this case, the rate of substitution increased following reassortment from 0.0027 to 0.0040 substitutions/site/year (Fig. S3E). The reassortment event labeled ‘8’ in Fig. 3B was an N2.2002B gene that changed pairing from H3.ClusterIVA to H1.Delta1A; for this event, the rate of substitution decreased after reassortment, from 0.0047 to 0.0032 substitutions/site/year (Fig. S3F). The reassortment event labeled ‘9’ in Fig. 3B was an N2.2002B gene that changed pairing from H1.Delta1a to H3.2010.1; for this event, the rate of substitution decreased following reassortment from 0.0031 to 0.0015 substitutions/site/year (Fig. S3G). The remaining reassortment events with onward transmission in the US swine population shown in Fig. 3 did not have a significant change in substitution rates following reassortment (Fig. S3).

**Figure 4.**
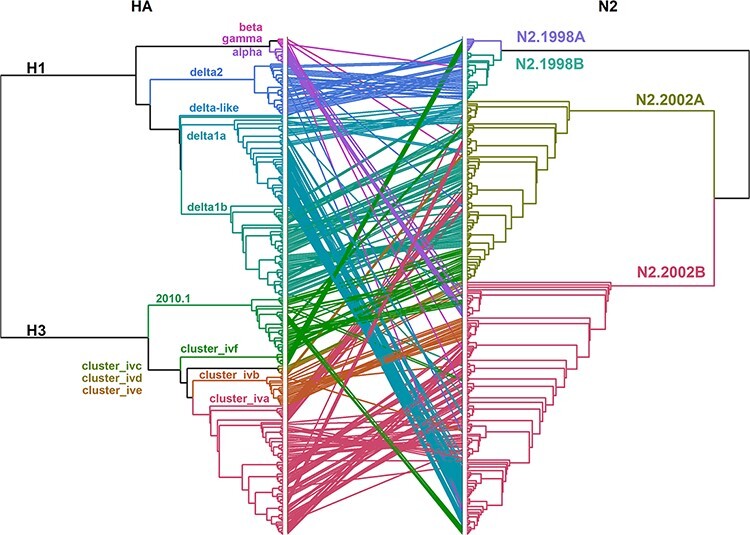
Tanglegram of the HA and paired N2 NA genes in US swine IAV. HA and N2 NA lineages are indicated by color and are presented on defining branches of the phylogeny. Connecting lines in the tanglegram are colored by the HA clade and demonstrate a link between HA and N2 present in the same virus. Maximum-likelihood trees are presented as cladograms with branch lengths scaled proportionally to the changes per site. Multiple, repetitive connecting lines between specific HA and N2 clades were suggestive of linkage disequilibrium. Reassortment is indicated when connecting lines move across multiple clades. For example, the H3.Cluster IVA and H1.Delta2 demonstrated connecting lines with multiple N2 clades, suggesting reassortment events occurred that subsequently became fixed in the swine population. Genetic divergence of HA was observed during these reassortment events based on the branch length.

A tanglegram consisting of a subsampled pairing of N2 and HA sequences was generated. Lines connecting N2 to their constituent HA within strain tended to be paired by clade, suggesting that these pairings were not random (Fig. 4). In Fig. 4, the connecting lines indicate NA–HA pairings: the frequency of detection and the topology of these trees demonstrated some pairing pattern shifts that subsequently became fixed in the population (Fig. 4). Prior to 2013, detections of H1.Delta1A were paired with N2.2002A. Following reassortment in 2013, this HA gene was frequently paired with N2.2002B and became the predominant H1.Delta1A pair by 2015 (Fig. S5A). Prior to 2011 H3.ClusterIVA were paired with N2.2002A, and after 2011 this HA was paired with N2.2002B and became the predominant pairing by 2013 (Fig. S5B). The H3.2010.1 was paired with the N2.2002A predominantly from 2014 to 2017, after which N2.2002B became predominantly detected (Fig. S5C).

**Figure 5.**
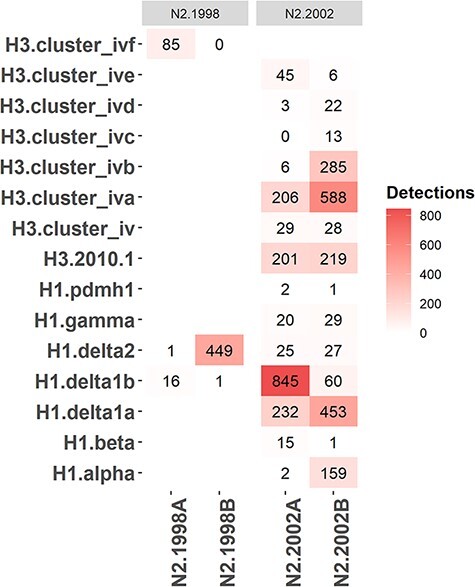
The observed detections of N2 neuraminidase (NA) clade and hemagglutinin (HA) genetic clade pairings in US swine from August 2009 to July 2018. More frequent N2–HA pairings are represented by red intensity, with raw detection data presented in the pairing.

The linkage disequilibrium observed in the tanglegram was statistically significant (chi-squared (*P* < 0.0001); Fisher’s exact test (*P* < 0.0001)), and the number of detections of distinct N2–HA pairings is presented in Fig. 5. N2.1998A was predominantly paired with H1.Delta1B and H3.ClusterIVF. This pairing demonstrated a temporal trend, as most of the N2.1998A detections were associated with the H1.Delta1B rather than H3.ClusterIVF following reassortment (Fig. 3). N2.1998B was almost exclusively paired with H1.Delta2. The N2.2002A was primarily paired with H1.Delta1B, but there were notable detections with H1.Delta1a, H3.ClusterIVA, and H3.2010.1 (Fig. 5). The N2.2002B was primarily paired with H1.Delta1A, H1.Alpha, and the H3.ClusterIVA-B (Fig. 5). The H3.2010.1 clade viruses were paired with either N2.2002A or N2.2002B in approximately equal numbers, but data collected over the last 2 years demonstrated an increase of N2.2002B pairing (Fig. S5C). The N2.1998B clade paired with the H1.Delta2 clade and the N2.2002A paired with the H1.Delta1 clade demonstrated near exclusive pairing (Fig. 5: chi-squared, *P* < 0.0001; Fisher’s exact test, *P* < 0.0001) and were analyzed to determine whether the relative genetic diversity of the paired genes was correlated. Detection of the H1.Delta2 paired with the N2.1998B first occurred in December 2009 and thereafter became the predominant N2–HA pairing of these clades. After 2012, the EPS of the N2.1998B and the H1.Delta2 became superimposed both temporally and in magnitude, suggesting that changes in the genetic diversity of these two genes are linked (Fig. S6A). Paired N2.2002A and H1.Delta1B demonstrated similar temporal aspects and magnitude of their respective measures of relative genetic diversity (Fig. S6B).

Similarly the relative genetic diversity of N2–HA pairs indicated that the substitution rates of N2 clades and paired HA clades are similar. Comparing the mean substitution rate per codon of N2 clades and select HA clades demonstrated similar substitution rates, N2.1998A: 0.0044, N2.1998B: 0.0046, N2.2002A: 0.0041, N2.2002B: 0.0045, H1.Gamma: 0.0047, H1.Delta2: 0.0049, H3.ClusterIVA: 0.0049, H3.2010.1: 0.0050 sites/substitution/year (Fig. S4). Collectively, the NA substitution rate was slightly lower than the HA substitution rate with a difference of 0.000475 sites/substitution/year, although the credible intervals did overlap, suggesting that these evolutionary rates are equivalent.

### 3.3 Diversifying selection and selective sweeps in the N2

An MEME demonstrated that multiple amino acid sites were under positive selection for each N2 clade (Table 2). The N2.1998A had four amino acid sites under positive selection in both the transmembrane and globular head regions. Although the analysis indicated position 414 as under positive selection, glycine was present at position 414 in all but three N2.1998A genes and is unlikely to be truly under positive selection. The N2.1998B had one transmembrane, five stalk, and four globular head sites under positive selection. There were two distinct monophyletic groups nested within the N2.1998B clade with the sites under positive selection also split across each clade, suggesting that these two groups diverged prior to 2009 (the earliest data used in our analysis). The N2.2002A had one stalk and three globular head sites under positive selection but no transmembrane sites. The N2.2002B had one transmembrane, one stalk, and seven globular head sites under positive selection. These data support the presence of positive selection across each of the N2 NA clades, indicating that functional amino acid substitutions are likely occurring in the gene as it is evolving.

**Table 2.** Amino acid sites detected as undergoing positive selection in the NA N2 gene of swine IAVs. Instances of episodic and pervasive selection were determined using an MEME in the HyPhy package. Sites under positive selection were also assessed with the FEL and SLAC methods in HyPhy.

	Site	Structural domain	Alpha	Beta^b^	*P* ^b^	*P* value	LRT	No. of branches	Amino acid polymorphisms
N2.1998A	23	Transmembrane	1.589	167.178	0.018	0.0479	4.54	1	I 91.3%, L 7.0%, F 0.9%, Y 0.9%
(*n* = 115)	147^a^	Globular head	0	11.394	0.309	0.0547	4.28	4	D 93.0%, G 4.3%, N 2.6%
	414	Globular head	2.024	20.691	0.113	0.0712	3.77	3	G 97.4%, S 1.7%, L 0.9%
	434	Globular head	0	51.322	0.075	0.0424	4.77	3	T 84.3%, A 14.8%, N 0.9%
N2.1998B	19^a^	Transmembrane	0	3.368	0.354	0.0435	4.72	7	A 76.4%, T 21.2%, G 1.1%, V 1.1%, S 2.2%
(*n* = 453)	40^a^	Stalk	0	1.312	1	0.0799	3.55	7	C 76.8%, Y 22.1%, S 0.7%, F 0.2%, H 0.2%
	66	Stalk	0	248.872	0.005	0.002	10.76	2	V 94.5%, M 5.3%, F 0.2%
	77^a^	Stalk	0	1.415	1	0.0797	3.56	9	K 77.9%, I 18.1%, E 0.7%, M 2.6%, N 0.2%, T 0.2%, V 0.2%
	82^a^	Stalk	0	1.509	0.646	0.0657	3.93	6	A 96.7%, S 1.8%, V 1.1%, T 0.4%
	86^a^	Stalk	0	1.225	1	0.087	3.39	8	N 81.2%, S 17.7%, T 0.7%, D 0.2%, R 0.2%
	149	Globular head	0	81.658	0.009	0.0077	8.11	2	F 78.6%, V 21.2%, C 0.2%
	269^a^^,^^b^	Globular head	0	2.943	1	0.0121	9.81	16	I 67.2%, L 20.6%, V 9.8%, M 1.3%, S 0.7%, R 0.4%
	313^a^^,^^b^	Globular head	0	1.873	0.997	0.0144	7.21	8	V 85.0%, I 6.2%, A 3.1%, T 2.2%, M 1.8%, D 1.3%, G 0.4%
	468^a^	Globular Head	0	0.879	1	0.0894	6.88	5	P 97.6%, L 0.7%, S 1.8%
N2.2002A	50^a^^,^^b^	Stalk	0	1.229	1	0.0267	3.34	6	A 73.8%, V 12.9%, T 11.3%, S 2.0%
(*n* = 256)	126^a^	Globular head	0	13.975	0.105	0.0282	5.57	7	P 95.7%, L 2.3%, S 0.8%, H 0.8%, R 0.4%
	127^a^^,^^b^	Globular head	0	1.262	1	0.0295	5.47	10	D 87.1%, N 7.8%, E 2.0%, G 2.0%, V 1.2%
	312^a^^,^^b^	Globular head	0.479	6.151	0.325	0.0798	3.56	7	I 87.1%, V 9.0%, T 2.0%, A 2.0%
N2.2002B	19	Transmembrane	0.583	324.387	0.006	0.0052	8.88	2	T 97.4%, S 1.3%, L 0.6%, A 0.3%, I 0.3%
(*n* = 310)	41	Stalk	0.946	41.509	0.023	0.0827	3.49	1	D 57.7%, N 41.6%, G 0.3%, S 0.3%
	199^a^^,^^b^	Globular head	0.518	4.964	1	0.0017	11.05	0	K 64.5%, E 20.3%, N 5.5%, G 5.5%, R 3.9%, Q 0.3%
	331	Globular head	0.656	27.71	0.026	0.0689	3.84	1	S 89.7%, R 8.7%, K 0.6%, N 0.6%, I 0.3%
	332	Globular head	0.944	213.11	0.006	0.0148	6.82	2	F 82.3%, L 17.1%, S 0.6%
	344^a^^,^^b^	Globular head	0	1.094	1	0.0467	4.59	5	E 84.8%, K 14.5%, R 0.6%
	399	Globular head	0.827	13.423	0.151	0.0627	4.02	13	G 42.6%, D 26.8%, E 24.5%, S 5.8%, K 0.3%
	402^a^	Globular head	0	0.877	1	0.0484	4.52	7	N 95.8%, D 3.2%, H 0.3%, K 0.3%, S 0.3%
	412^a^	Globular Head	0	1.629	0.599	0.0514	4.4	7	V 71.9%, I 28.1%

a Sites common between MEME and FEL.

b Sites overlapping between MEM and SLAC.

Sweep Dynamics plots for each major genetic clade indicated that certain amino acid sites transitioned within the population over time (Table 3). While the analysis did not find any specific position significant using Fisher’s exact test, the visualization shows multiple transitions that appear to be linked, with these positions changing in frequency across the study period (Fig. S7). Amino acid mutations were visualized across the phylogenetic backbone of each N2 genetic clade using Nextstrain (Fig. S8–S11), with sites identified in Sweep Dynamics plots annotated. Additionally, substitutions at positions 12, 27, 29, 48, 79, 135, 147, 185, 243, 249, and 298 were observed in two or more NA clades (Table 3, Fig. S7).

**Table 3.** Detection and amino acid location of selective sweeps within N2 NA genetic clades following reassortment. Amino acid position and identity in the N2 gene is indicated where the detected amino acid shifted by greater than 50 per cent frequency within each NA clade.

NA clade	Transition
N2.1998A	S43N, N86I, N141S, D147G, V165I, T238A, I254V, I265T, V307I
N2.1998B	V13I, I17L, T19A, V30I, Y40C, N43H, N47T, I62T, E64K, K64N, I77K, K93R, D147N, V149F, I165V, K187R, E199N, K199E, M241V, R249I, I263V, L269I, V313A
N2.2002A	L22F, N41D, V50A, M51I, K75R, D86S, K93N, D127G, I149V, S161N, I176M, I194V, D199N, F205L, I257V, V263I, I263V, R264H, K267T, S284F, D309N, H310Y, V312I
N2.2002B	I26V, V26I, D41N, N43D, Q49H, L52F, K62T, I73V, L81P, A82T, I257V, I263V, I312T

### 3.4 Reassortment and permissiveness of N2 genes to HA pairings

Reassortment resulting in HA and N2 gene pairings did not appear to be random (Fig. S12). There was limited reassortment of the N2.1998A clade NA genes from H3.ClusterIVF to H1.Delta1B viruses (Fig. S12). The N2.1998B clade genes were almost exclusively paired with H1.Delta2, with very few sporadic dead-end reassortment events detected. The few reassortment events within the N2.1998B clade were given high and uniform posterior probabilities as well as Bayes factors, but despite N2.1998B reassortment, it was not consistently detected or sustained in swine. The N2.2002A genes demonstrated reassortment from H1.Delta1A, H1.Delta1B, H3.ClusterIVA, and H3.2010.1 to multiple other HA clades, indicating the N2.2002A genes may pair with multiple HA genes. The N2.2002B demonstrated the greatest breadth of reassortment with the gene pairing with H1.Delta1A, H1.Alpha, H3.ClusterIVA, H3.ClusterIVB, and H3.2010.1. These results indicate that some NA genes are permissive of multiple HAs (i.e. approaching random), whereas other NA genes were only detected with specific HA genes. This Bayesian approach is consistent with the clade pairing patterns observed in the tanglegram and chi-squared analysis (Figs 4 and 5).

### 3.5 Spatial dissemination of N2 genetic clades

The spatial detection of N2 clades from 2009 to 2018 indicated differences in geographic distribution (chi-squared test *P* < 0.0001: Fig. S2, S13) that reflect general patterns of swine agriculture in the USA. Viruses containing N2.1998A had the fewest detections and had limited geographic distribution in the Midwest, primarily in Iowa or adjacent states. The N2.1998B was detected most frequently in the Southeast (North Carolina), with a few detections in the Midwest, including Indiana and Iowa. The N2.2002A was detected most frequently in the Midwest and was rarely detected in other regions of the USA. The N2.2002B was detected in the Midwest as well as the Southeast (North Carolina). On a US state basis, differences in the detection of viruses containing specific NA clades were observed: for example, viruses detected in Iowa contained NA clades in a descending order of frequency, N2.2002B, N2.2002A, and then N2.1998B.

**Figure 6.**
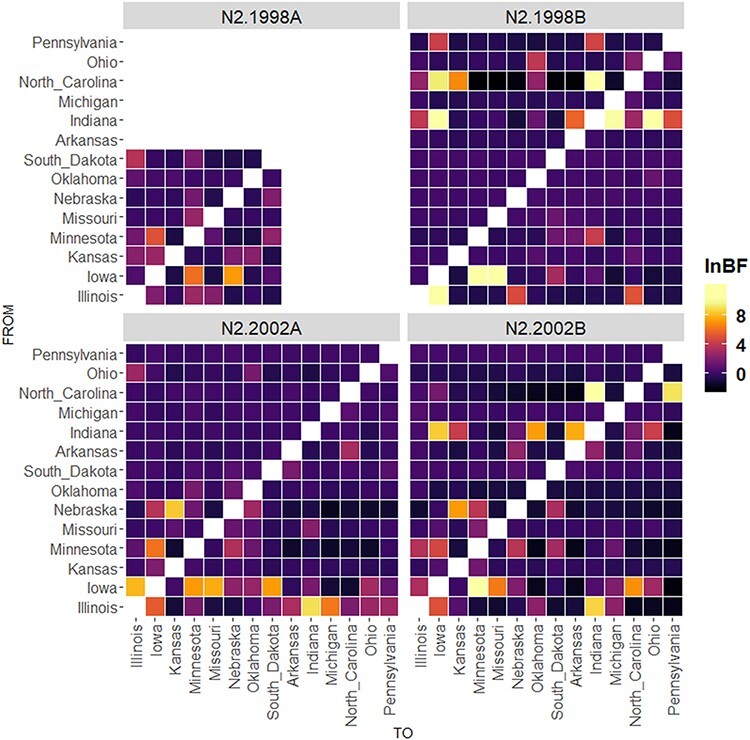
Bayes factors for inferred state-to-state movement of N2 NA clades demonstrating the frequency with which particular genetic clades are moving from particular US locations on the *y*-axis to other states on the *x*-axis.

The interstate N2 gene movement, inferred by Bayesian phylodynamic techniques (Fig. 6), demonstrated that even infrequently detected N2 had geographic movement despite the low overall detections that are presented in Fig. S13. IAV containing N2.1998A moved between Minnesota, Iowa, and Nebraska. IAV with N2.1998B moved from North Carolina into the Midwest as well as from Iowa to Minnesota and Missouri. Despite movement to the Midwest, the N2.1998B was not frequently detected in that region. IAV with N2.2002A were moved (likely transported with live swine) from Iowa and Indiana to multiple locations throughout the Midwest, including Nebraska and Kansas. IAV with N2.2002B moved from North Carolina to Indiana and Pennsylvania, with Indiana, Iowa, and Illinois also acting as sources of N2 gene distribution. Nebraska appeared as a possible source for N2.2002B to move into Kansas. Despite the inference of transmission of almost all N2 genetic clades to all geographic regions across the US, the detection of N2 clades within surveillance data suggested that many of these clades do not persist.

## 4. Discussion

Multiple cocirculating clades of swine IAV N2 were reported, with a significant amount of genetic and antigenic diversity that challenges control efforts (Anderson et al. 2013; Zeller et al. 2018a; Kaplan et al. 2021). Our data demonstrated that diversity in the N2 gene has increased dramatically over the past 10 years. The previously described N2.1998 lineage genetically diverged in 2007.6 (2006.5–2008.8, 95 per cent HPD) into the N2.1998A and N2.1998B statistically supported monophyletic clades. The previously described N2.2002 lineage genetically diverged in 2006.0 (2004.9–2007.1, 95 per cent HPD) into the N2.2002A and N2.2002B statistically supported monophyletic clades. Our data suggested that this observed diversity has occurred due to the concurrent forces of antigenic drift, reassortment that resulted in novel NA–HA pairings, and interstate movement of IAV. These dynamics played a critical role in establishing predominant NA–HA pairings. The interstate movement of IAV with pigs may perpetuate the cycle of antigenic drift, reassortment, and diversification through the regular introduction of novel NA–HA pairings to different regions; and this creates significant challenges to the formulation of well-matched vaccines and other control efforts.

Viruses containing N2.1998 and N2.2002 lineage genes had multiple reassortment events, leading to the emergence of genetically distinct N2 clades. Our data demonstrated that N2 genes may persist at low detection levels, exchange HA genes via reassortment, and then through diversification and spatial dissemination the ‘minor’ clades may become predominant. This is best exemplified by the frequency of detection of the N2.1998B and H1.Delta2 genes. The H1.Delta2 HA gene was originally paired with an N2.2002 when first detected in the US swine population, and this combination occurred at relatively low levels prior to 2011. In late 2011, a reassortment event that resulted in the pairing of the N2.1998B with the H1.Delta2 HA occurred; this coincided with correlated increases in the relative genetic diversity of both the H1.Delta2 and N2.1998B (Fig. S6A) and subsequent substantive increases in the number of detections of the N2.1998B gene, with this combination representing 16 per cent of H1 detections in 2017 (Zeller et al. 2018a). Similarly, the N2.1998A clade persisted when paired with the H1.Delta1B HA, whereas the original pairing with an H3.ClusterIVF HA became infrequently detected. It is reasonable to suggest that reassortment could result in the diversification of both HA and NA genes, adaptation, and given that swine regularly move across the USA, the potential for widespread dissemination of novel gene pairings.

An increase in detection frequency of specific NA–HA pairs following reassortment may be related to a favorable balance between the NA and HA protein activity (Hensley et al. 2009), a better match between the surface genes and internal gene segments for replication (Gao et al. 2017), or novel antigenic properties that evade population immunity (Abente et al. 2016). The tanglegram and our quantification of pre- and post-reassortment substitution rates indicated that there could be changes in evolutionary trajectory following reassortment, although this appeared to be NA–HA gene dependent. This was evidenced in the complete replacement of one NA–HA pairing with another over time, and that there were significantly different substitution rates in four of the nine reassortment events, although the pattern was not a consistent (i.e. for two pairings substitution rate increased and for two it decreased). We speculate that newly paired HA and NA genes require adaptation. Notably, an additional collateral consequence of reassortment (antigenic shift) may be an increase in antigenic drift in both surface proteins. Although we did not empirically quantify virus phenotype, we demonstrated that there were linked amino acid substitutions within each N2 clade that occurred during the study period, and some of these mutations were within important functional sites (Tables 2 and 3). These data highlight the necessity to monitor genetic changes in both NA and HA and patterns of NA and HA pairings through IAV surveillance in swine. Our data suggest that NA–HA gene pairings have different evolutionary rates, and novel gene pairings may result in linked substitutions across the N2 gene that could impact vaccine formulations.

Our data revealed linkage disequilibrium between NA and HA genes. We detected reassortment events that were single (dead-end) detections or followed by multiple detections. The increased frequency of detection of novel NA–HA pairs following reassortment was indicative of sustained transmission of that NA–HA pairing within the swine population. Some reassortment events with subsequent transmission were also characterized by subsequent increased genetic diversity of both the N2 and HA, indicated by increases and decreases in relative genetic diversity across an 8-year period. The N2.1998B is of particular interest as it was almost solely paired with the H1.Delta2 clade, despite detections of reassortment with other HA that did not appear to persist. The loss of these pairings from the swine population may be due to ecological (e.g. swine production system specific IAV circulation), virus (e.g. NA and HA protein activity balance), or host immune factors (e.g. antigenic properties) that require additional study. A possible explanation for the diversity changes we document is that the activity of these two proteins requires a balance for an IAV infection that results in transmission (Mitnaul et al. 2000; Das et al. 2011). The increase in diversity may arise due to selection pressure to optimize the HA and NA balance, evidence for this is a lag before the increase in detection numbers following reassortment, i.e. a novel NA and HA requires a period of adaptation (detected as an increase in relative genetic diversity) prior to increasing in detection frequency (Fig. S6). Additionally, we demonstrated that some sites in each N2 NA clades were under positive selection and some of these were in locations that have functional significance. We are unable to determine whether selection at these sites is the result of immune-driven selection (e.g. Su et al. 2015) or a consequence of reassortment and the emergence of novel gene pairings. Additional experiments could address this issue through deliberate manipulation of NA–HA gene pairings and subsequent sequencing during infection and transmission studies (Dinis et al. 2016; Moncla et al. 2016). Despite the limitations of our approach, we do demonstrate positive selection within functional areas of the gene, and when mapping amino acid substitutions across the phylogeny, some substitutions appear to be associated with reassortment (Figs S7–S11).

The genetic evolutionary rates of the N2 NA and the HA genes in swine were shown to have similar rates. In a limited number of studies, the substitution rate for the HA has been consistently similar to the NA across studies when measured empirically (Saitou and Nei 1986; Air et al. 1990) and computationally (Khandaker et al. 2013; Westgeest et al. 2014). However, the general paradigm in IAV biology is a focus on the evolution of the HA and the role of immune-driven selection in its dynamics. Our data fit the growing body of literature that suggest that the NA evolves in a similar manner to the HA and that immune-driven selection may lead to functional amino acid changes (Correia, Abecasis, and Rebelo-de-andrade 2018). We detected 26 amino acid sites that demonstrated a signal of diversifying selection; these included sites that were pervasively observed across different clades, some of which may have functional consequences. However, relative to data on selection analyses in HA genes, the number of sites in the NA undergoing diversifying selection is more limited, possibly as a compensatory mechanism for genetic changes in the HA.

The four monophyletic N2 clades circulating in US swine had different geographic patterns and frequencies of reassortment. The N2 clades that were paired with more HA clades were the N2.2002A and N2.2002B clades, and these both had a higher relative genetic diversity when compared to the N2.1998A and B clades, as well as being frequently detected over the study period. This observation may reflect a ‘sampling effect’ where a larger pool of genetic diversity in the N2 increases the likelihood of a reassortment event with an HA that may demonstrate onward transmission. There were also strong regional patterns in the number of N2 clades detected. The N2.1998A was limited to three states in the Midwest, while the majority of N2.1998B detections were in the Southeast (North Carolina). The N2.2002A was detected across the Midwest and the N2.2002B was detected in the Midwest and Southeast. The data used in this study are derived from a passive surveillance program where clinically sick pigs may be sampled, and if IAV positive, the specimen may be sequenced (Anderson et al. 2013). Consequently, these data are geared primarily to address diagnostic questions, with the benefit of providing a snapshot of the genetic diversity of IAV circulating in US swine. A notable weakness in our data is the inability to measure core epidemiological parameters (e.g. prevalence) and that states with large swine populations and their genetic N2 clades are likely overrepresented in the dataset. However, despite these limitations, our inference is derived from patterns occurring across over 8 years’ worth of data, and this supports general swine agricultural practices where the Midwest appears to be a central point for pig shipment. Prior research suggested that the transmission of IAV in swine was observed in directed patterns in the USA, namely from the Southwest and Southeast to the Midwest (Nelson et al. 2011), and our data on the movement of the N2 genes are in general agreement with these prior findings. The implication of this is that directed movement of viruses could influence the opportunity for reassortment and subsequent patterns of diversity: areas that largely receive pigs and their viruses may provide more opportunities for reassortment. Consequently, a location such as Iowa would be expected to have the highest IAV genetic diversity as well as the most reassortment events. However, there are exceptions; some N2 clades remained geographically restricted despite interstate movement. The N2.1998B was introduced to the Midwest multiple times and to multiple states but was not successfully established, indicating that factors (e.g. genetic compatibility or host-related) have prevented this clade from expanding across the USA.

## 5. Conclusion

This study demonstrates linkage disequilibrium of NA and HA of the influenza virus in swine in the USA that likely reflects differences in fitness and the coordination of the activity of these proteins. While this study focused on the N2 due to its frequency of detection and apparent reassortment and genetic diversity, understanding the evolution of N1 and N2 subtypes will benefit vaccine development. If the HA vaccine antigen does not match currently circulating strains, a vaccine that contains a matched NA antigen may still reduce clinical signs and shedding of the virus. Further, monitoring the NA may serve as an early indicator of phenotypic change based on detected reassortment events. IAV reassortment may increase antigenic drift (Vijaykrishna et al. 2011), and the current movement of swine between geographic locations in the USA may increase these events. Future surveillance efforts with increased monitoring and surveillance of the genetic diversity of NA will complement HA surveillance and efforts to protect swine from influenza and reduce the chance for zoonotic transmission.

## Supplementary Material

veab090_Supp
