# Supplementary material for: Spatial and temporal coevolution of N2 neuraminidase and H1 and H3 hemagglutinin genes of influenza A virus in US swine
Source: Virus Evol. 2021 Oct 8;7(2):veab090. doi: 10.1093/ve/veab090 (PMC8864744; doi:10.1093/ve/veab090)
Supplement: veab090_Supp [file veab090_supp.zip › Zeller_etal_N2_supFigure-R2.pdf]

SUPPLEMENTARY MATERIAL

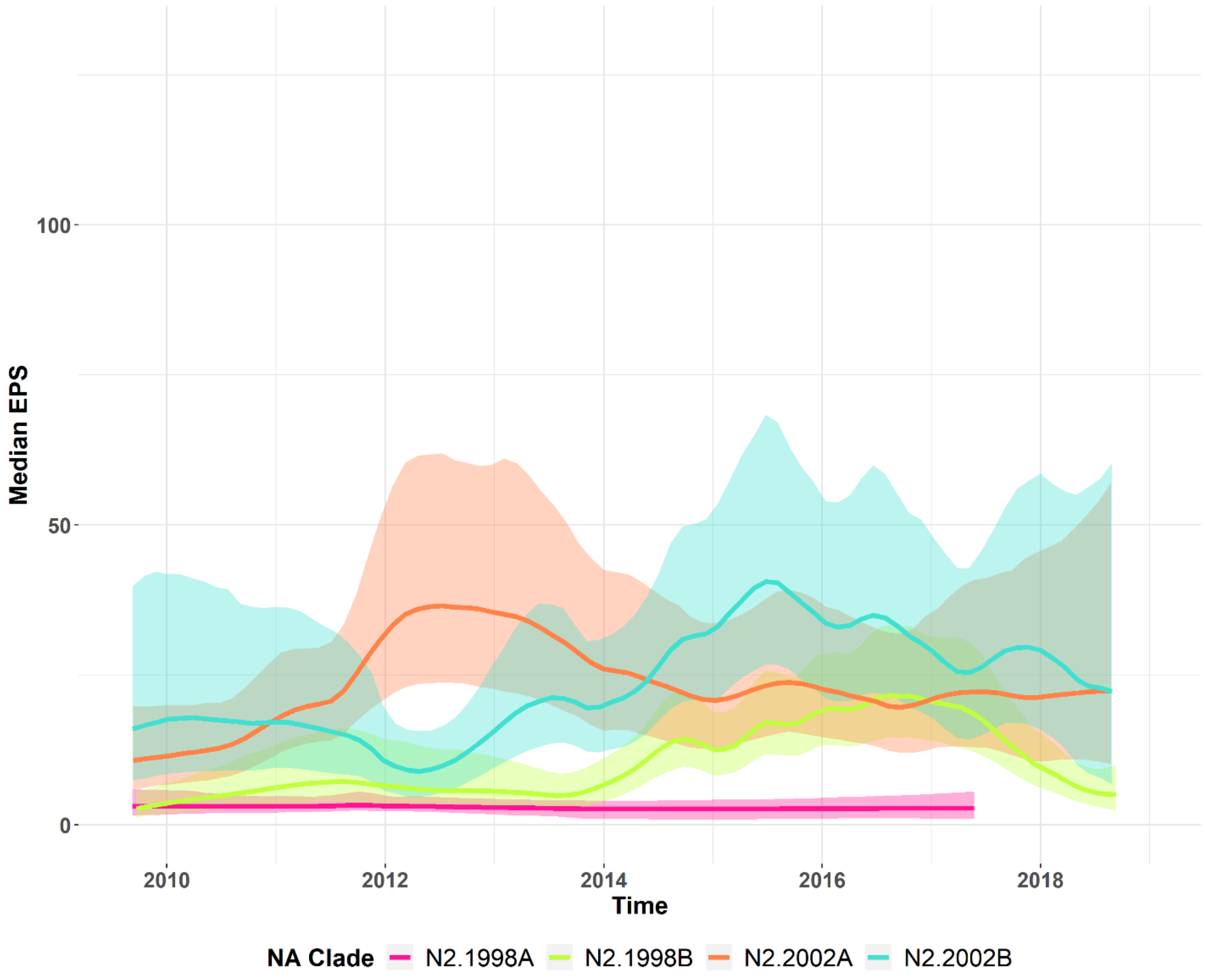

Figure S1. Relative genetic diversity, measured as effective population size (EPS), of the N2 clades detected in North American swine from 2009-2018, N2.1998A, N2.1998B, N2.2002A, and N2.2002B. Temporal dominance in relative genetic diversity varied between statistically supported monophyletic clades of NA N2. N2.2002A genetic diversity peaked in mid-2012, N2.2002B peaked in mid-2015, and N2.1998B peaked mid-2016. N2.2002A and N2.2002.B clades were maintained at a moderate genetic diversity across the study period.

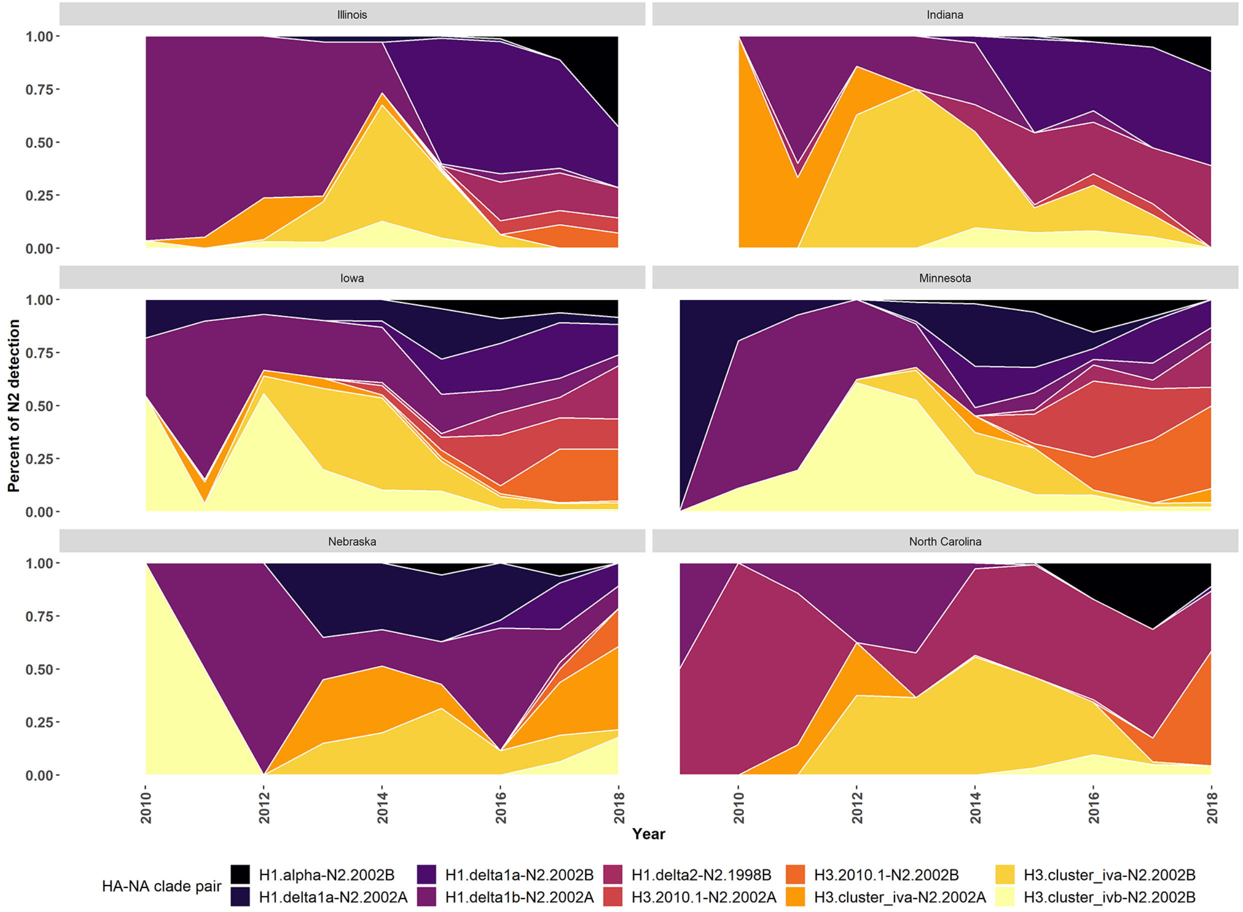

Figure S2. The top ten pairings of hemagglutinin (HA) and neuraminidase (NA) and their percent detection per year from August 2009 to July 2018, subsetted by U.S. state of detection. The top six states were selected for visualization as they had greater than 250 N2 detections per state.

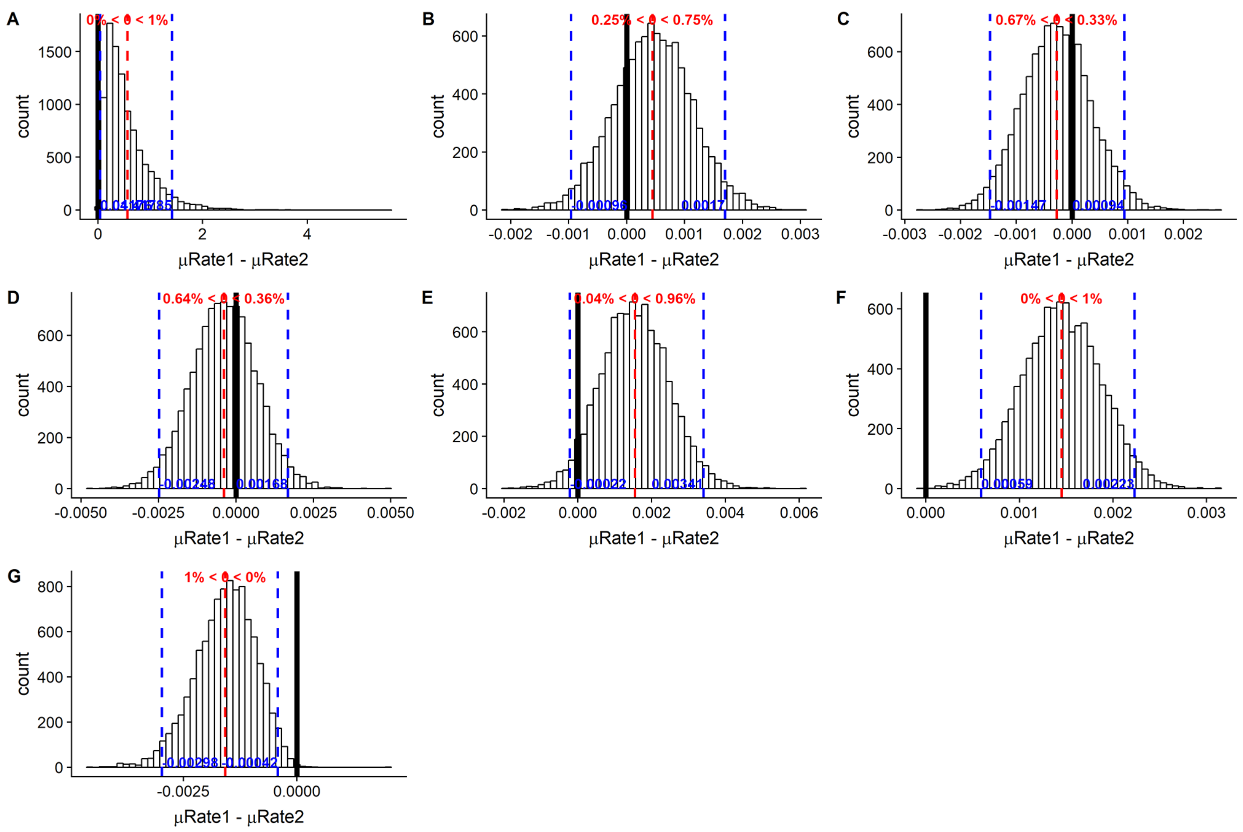

Figure S3. Subtraction of the distribution of the parameter of mean substitution rate on Bayesian analysis partitioned by neuraminidase (NA) from a donor hemagglutinin (HA) to recipient HA. Clock and substitution models were unlinked for these analysis. (A) IAV containing N2.1998A paired with H3.ClusterIVF donating NA genes to IAV with H1.Delta1B (Figure 2, reassortment event 1), (B) N2.2002A paired with H1.Delta1B donating NA genes to H3.2010.1 (Figure 2, reassortment event 4), (C) N2.2002A paired with H3.ClusterIVB donating NA genes to IAV with H1.Alpha (Figure 2, reassortment event 5), (D) N2.2002A paired with H1.Alpha donating NA genes to IAV with H3.2010.1 (Figure 2, reassortment event 6), (E) N2.2002B paired with H3.ClusterIVB donating NA genes to IAV with H3.ClusterIVA (Figure 2, reassortment event 7), (F) N2.2002B paired with H3.ClusterIVA donating NA genes to IAV with H1.Delta1A (Figure 2, reassortment event 8), (G) N2.2002B paired with H3.ClusterIVA donating NA genes to IAV with H1.2010.2 (Figure 2, reassortment event 9). The mean of the parameter is indicated by a dashed red line. The 95% higher posterior density is marked between the two blue dashed lines and represents a credible interval. Zero is represented with a thick black line. The presence of zero within the credible interval indicates a practical equivalence between the two mean substitution rates, as seen in B, C, and D. Zero being outside the credible interval represents a difference between the two mean substitution rates, as shown in A, E, F, and G.

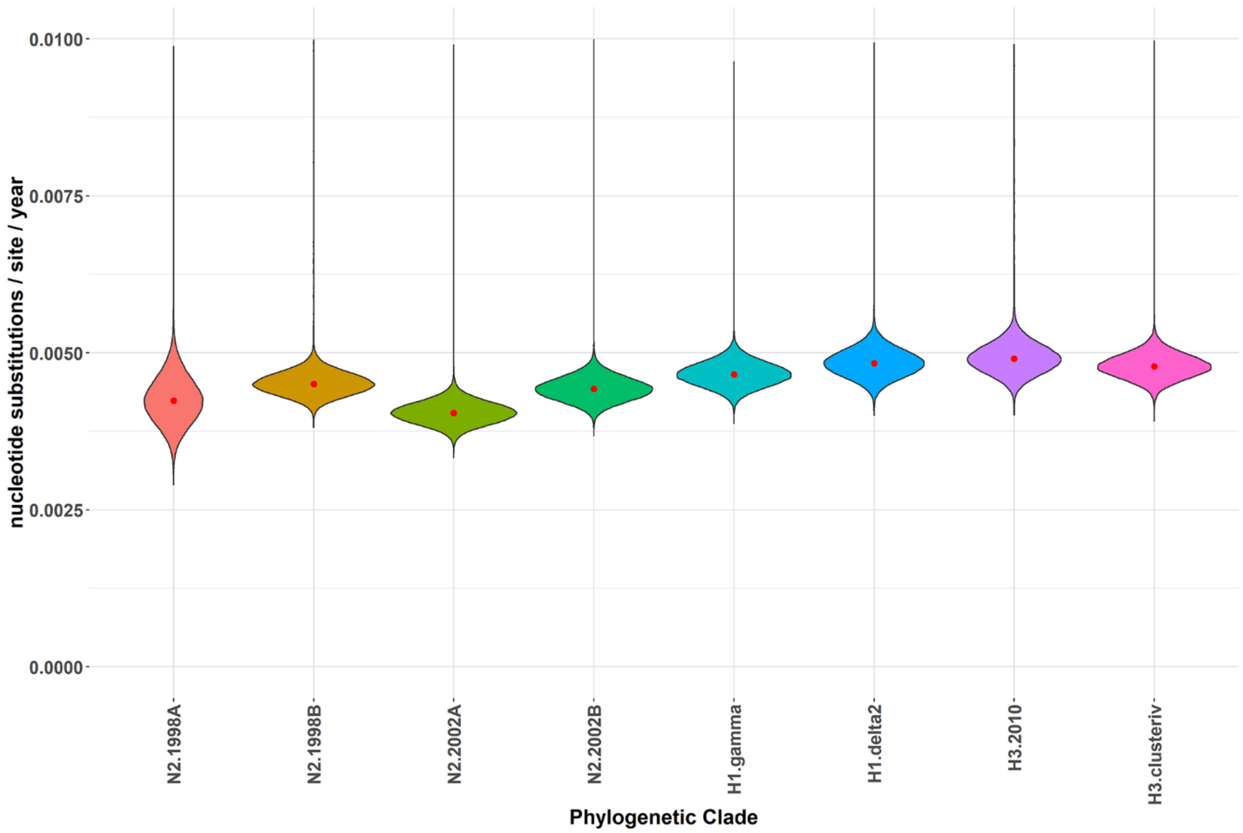

Figure S4. The mean nucleotide substitution rate of H1 and H3 hemagglutinin and N2 neuraminidase clades. Substitution rates were calculated using BEAST2. Each codon position represented a different log normal relaxed clock model, and a GTR+ Γ substitution model with four categories. The three clock rates for each codon position were averaged to determine the mean nucleotide substitution rate across the entire gene. N2.2002A had the lowest mean substitution rate at 0.0041, while H3.2010.1 had the highest substitution rate at 0.0050. The mean HA substitution rate was 0.0005 nucleotides/site/year faster than the NA mean substitution rate.

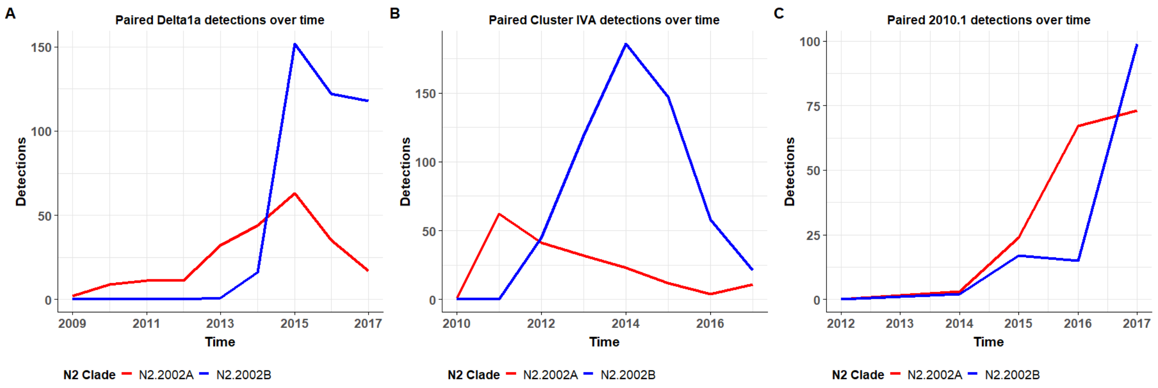

Figure S5. Neuraminidase clades over time paired with (A) H1.Delta1a, (B) H3.ClusterIVA, (C) H3.2010.1 in IAV detected from 2009-2018. Graphs depict the number of detections of each HA and NA pairing. In these three examples, there was a change in predominant HA and NA pairings over time.

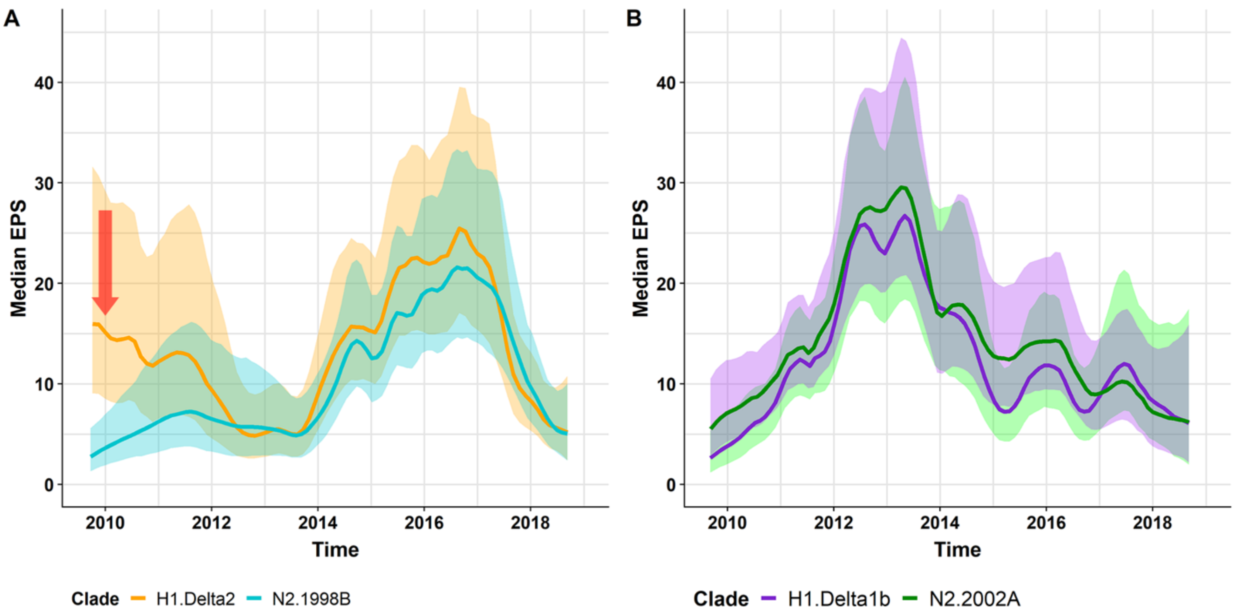

Figure S6. (A) Relative diversity of the N2.1998B clade and the H1.Delta2 clade over time from 2009-2018. Relative diversity of H1.Delta2 and N2.1998B was not correlated until after 2012. H1.Delta2 was prior paired with N2.2002, with the first detections of H2.Delta2-N2.1998B occurring in December of 2009, denoted by the red arrow. (B) Relative diversity of the N2.2002A clade and the H1.Delta1B clade that were paired from 2009-2018. Median EPS were denoted by lines with the 95% higher posterior density shaded in the same color. Temporally matched changes at similar magnitudes suggested a correlation between the diversity of the shown NA-HA

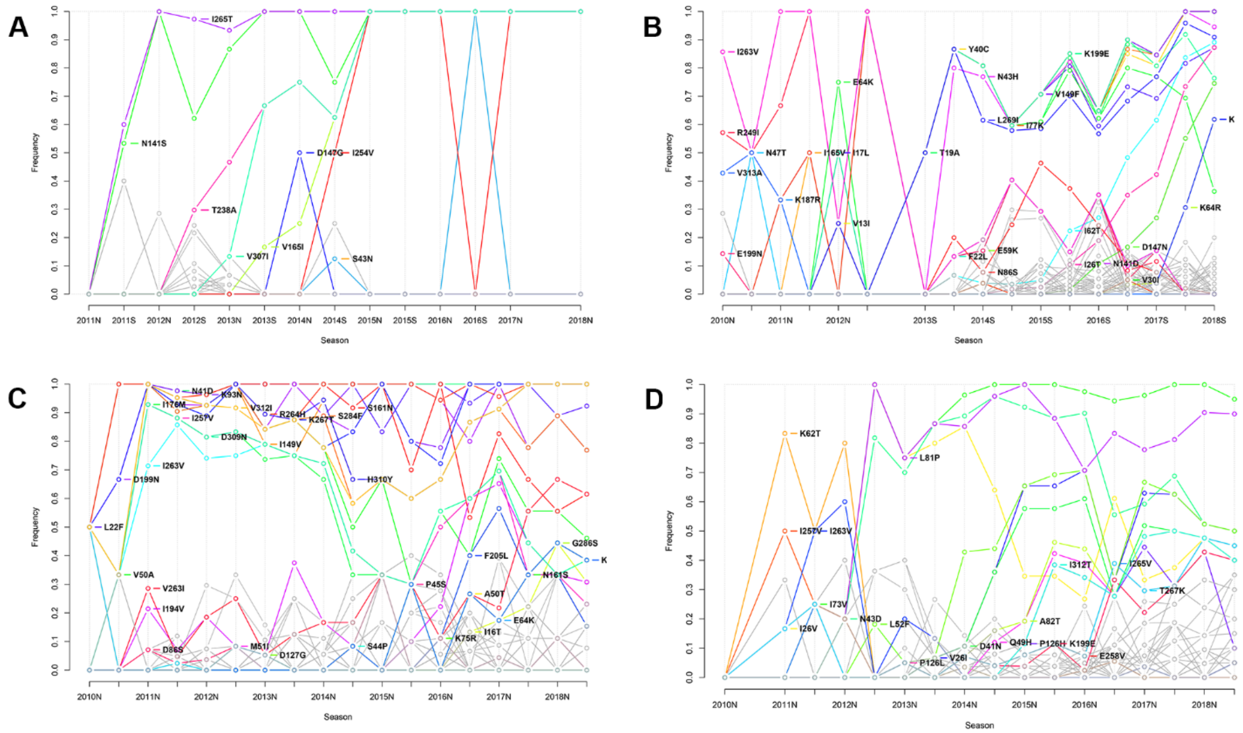

Figure S7. Frequency of amino acids detected at specific positions over time of (A) N2.1998A, (B) N2.1998B, (C) N2.2002A, (D) N2.2002B determined by Sweep Dynamics plots. Detections that cross a threshold of 50% of the population in a season are colored.

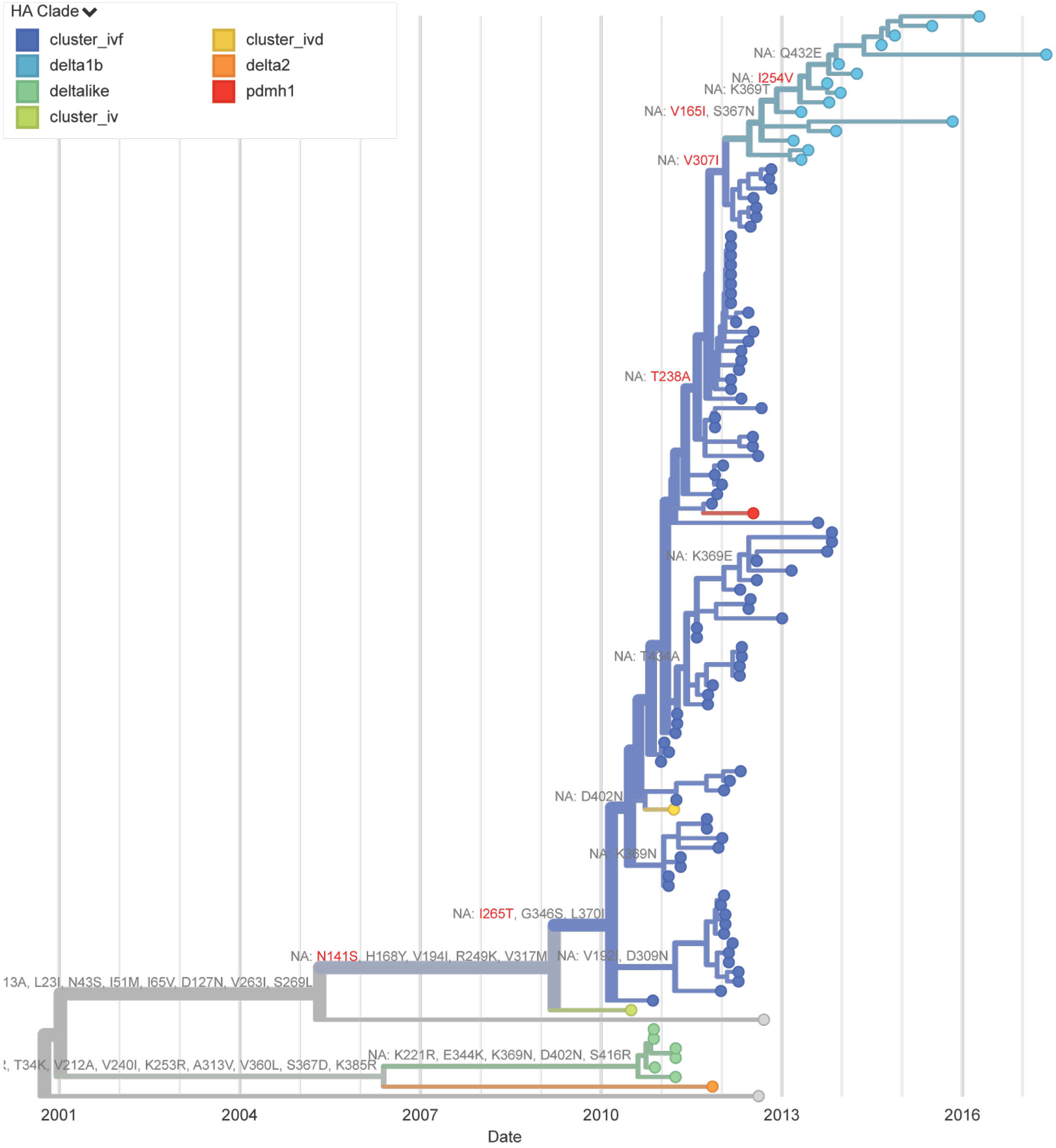

Figure S8. Time scaled phylogenetic tree of N2.1998A rendered by Nextstrain. Leaves are colored by paired HA clade, and tree backbone is annotated by inferred amino acid mutations. Mutations identified by the Sweep Dynamics plots are highlighted in red.

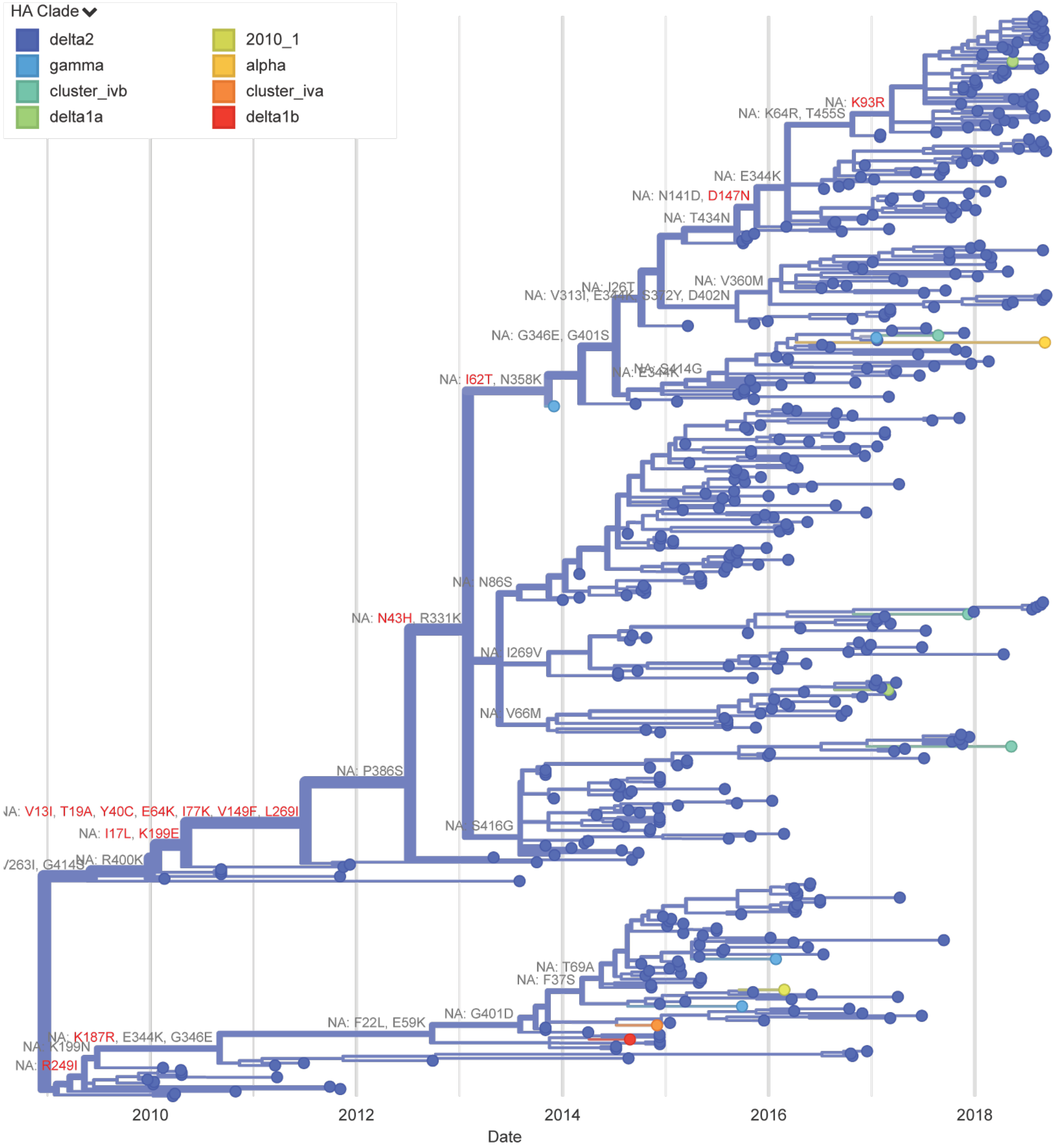

Figure S9. Time scaled phylogenetic tree of a subset of N2.1998B sequences rendered by Nextstrain. Leaves are colored by paired HA clade, and tree backbone is annotated inferred amino acid mutations. Mutations identified by the Sweep Dynamics plots are highlighted in red.

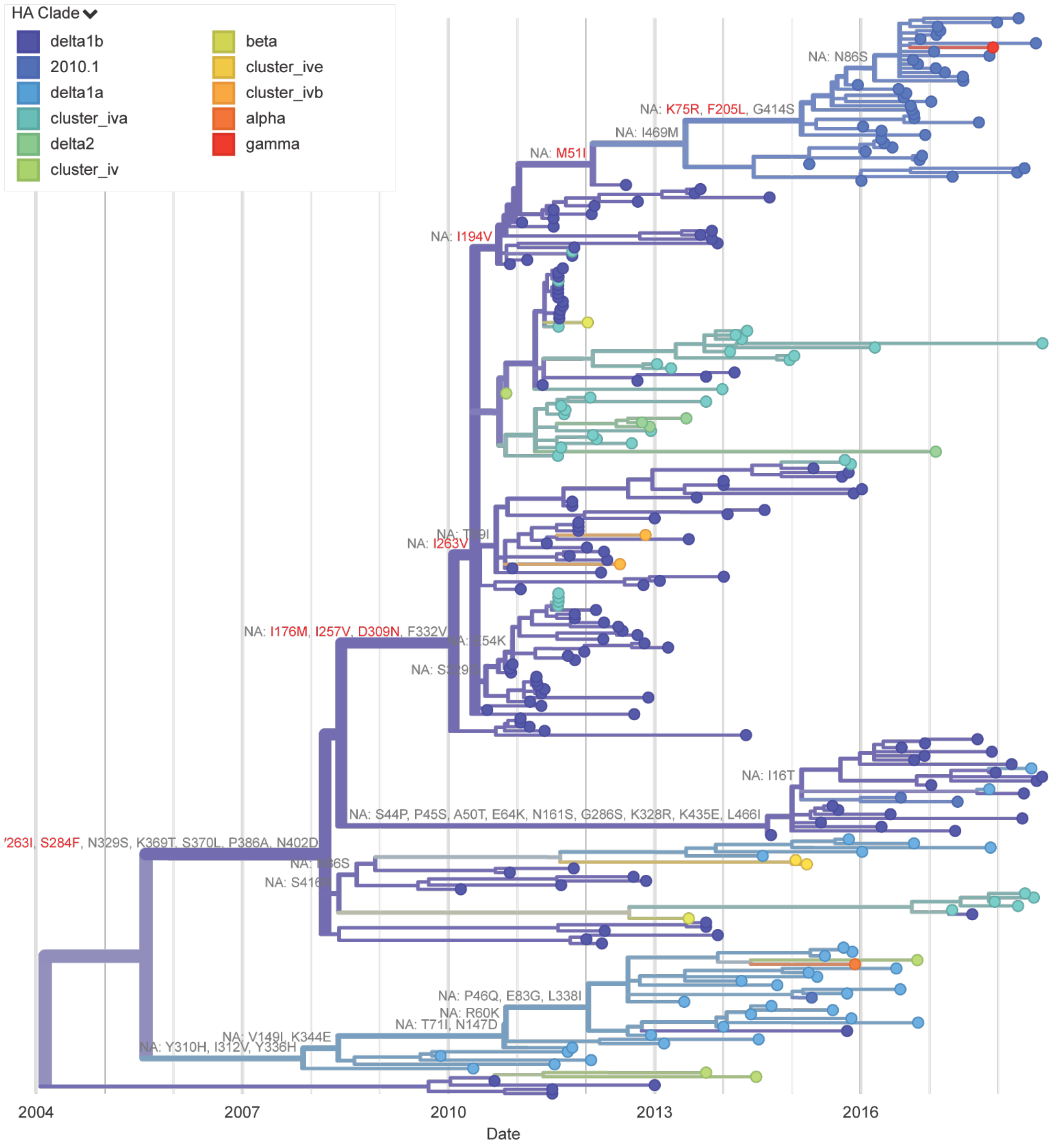

Figure S10. Time scaled phylogenetic tree of a subset of N2.2002A sequences rendered by Nextstrain. Leaves are colored by paired HA clade, and tree backbone is annotated by inferred amino acid mutations. Mutations identified by the Sweep Dynamics plots are highlighted in red.

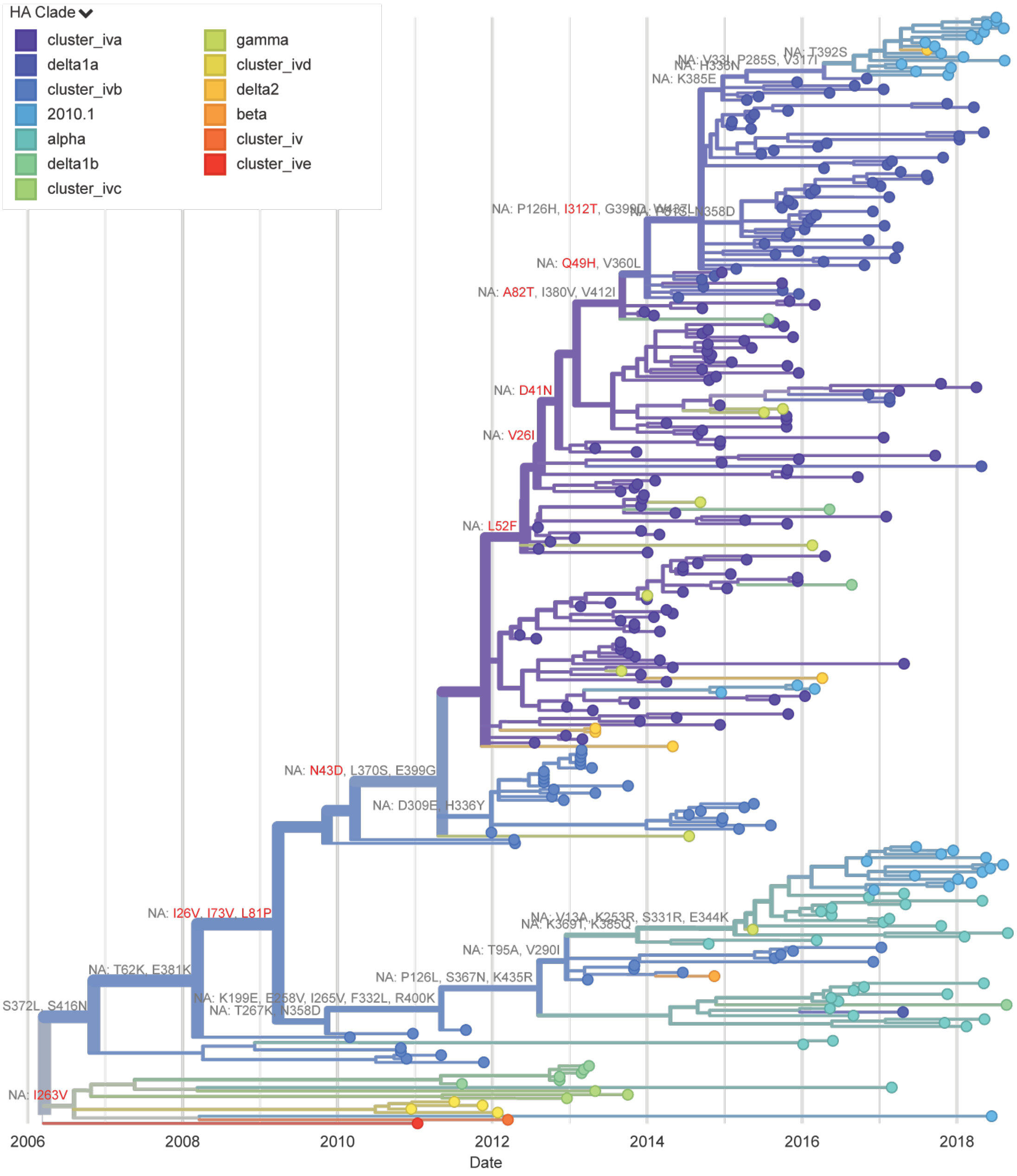

Figure S11. Time scaled phylogenetic tree of a subset of N2.2002B sequences rendered by Nextstrain. Leaves are colored by paired HA clade, and tree backbone is annotated by inferred amino acid mutations. Mutations identified by the Sweep Dynamics plots are highlighted in red.

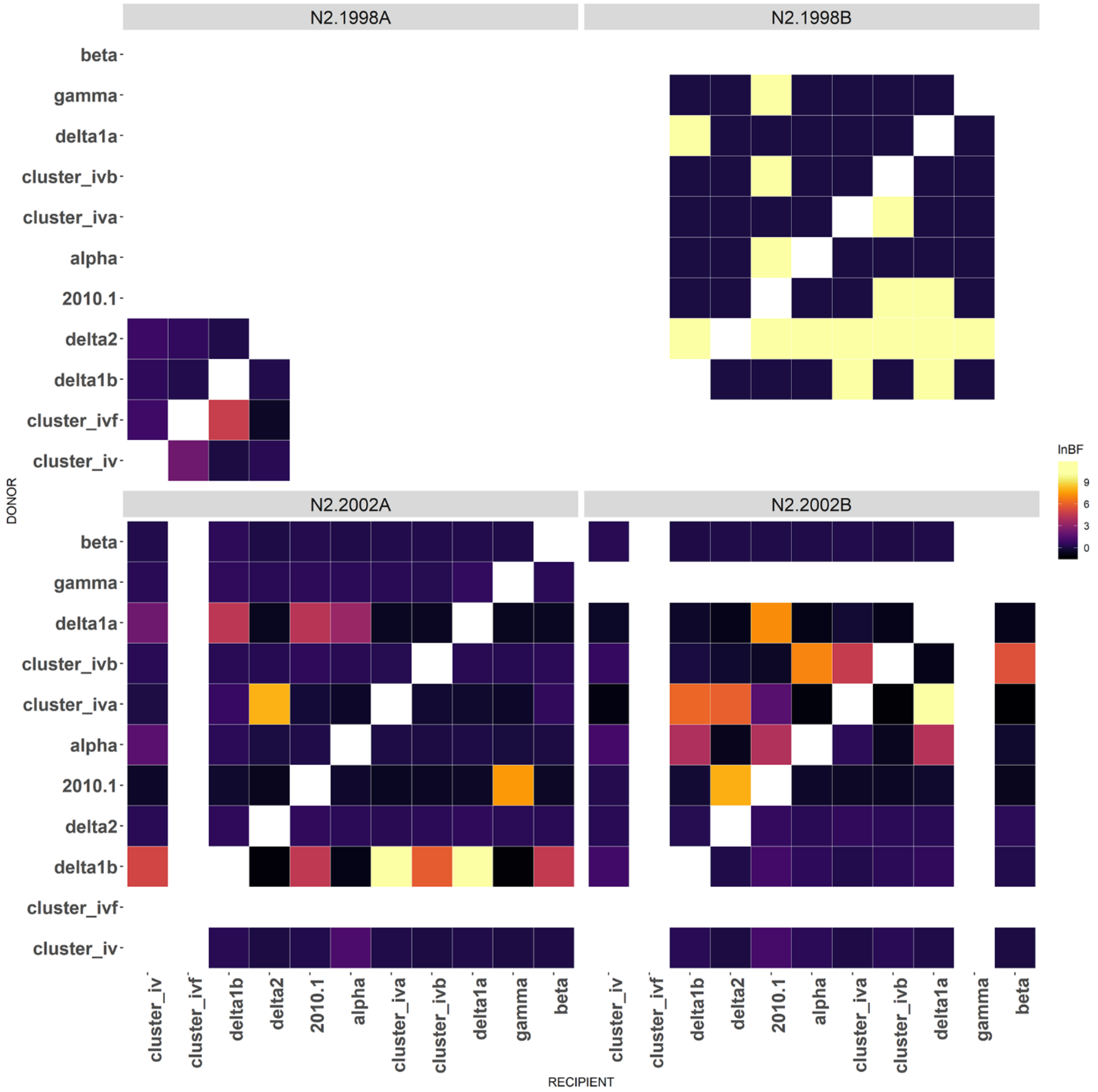

Figure S12. Bayes factors (Ln BF) representing the confidence of transition events of the hemagglutinin (HA) gene by clade for neuraminidase (NA) gene by N2 clades from 2009 to 2018. Higher Ln Bayes factors represent in warmer colors are higher evidence for reassortment; uncolored squares present an absence of the transition between genes. The N2.1998A clade demonstrated the least frequent reassortment of the N2 clades, but there was a transition from H3.ClusterIVF to H1.Delta1B. The small numbers of detected reassortment events for N2.1998B had strong support due to near exclusive pairing between N2.1998B and H1.Delta2. Both N2.2002A and N2.2002B NA genes demonstrated evidence for multiple reassortment events.

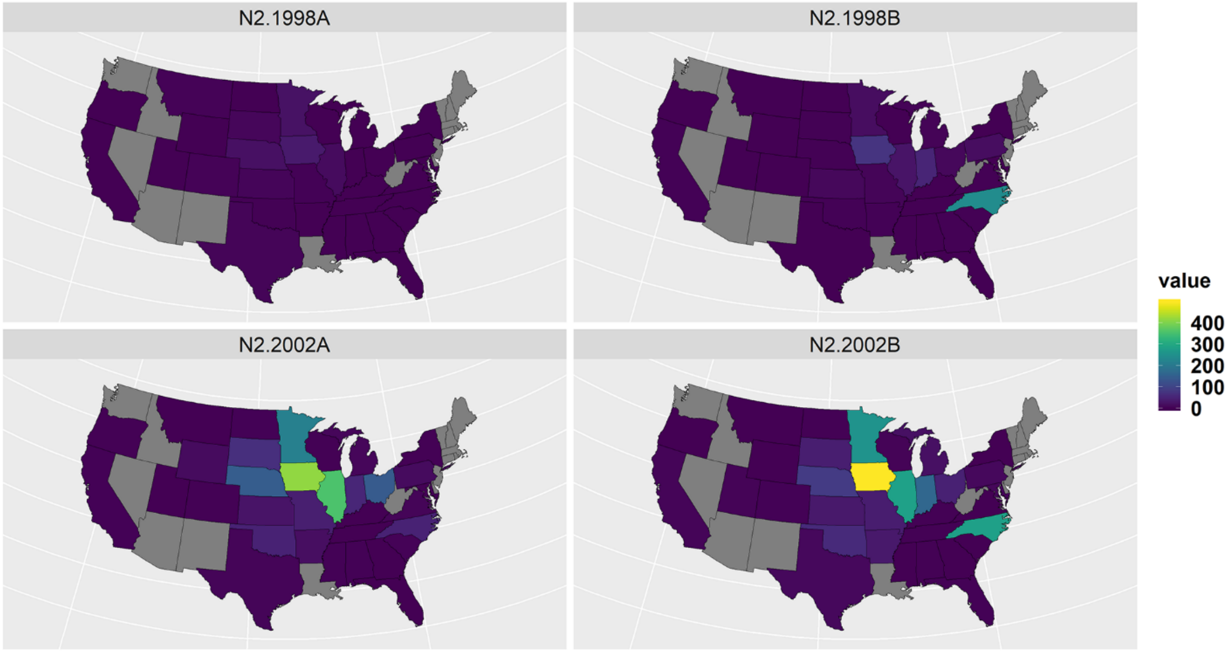

Figure S13. (A) Geographic representation of each N2 clade from 2009-2018. N2 clade was dependent on location (chi squared: $p < 0.001$). N2.1998A was observed with few detections in limited locations in the Midwest. N2.1998B is detected primarily in North Carolina, with few detections present in the Midwest including Indiana and Iowa. N2.2002A was detected in the Midwest but was rarely detected in North Carolina. N2.2002B was detected in the Midwest as well as North Carolina.
